# Adverse Obstetrical Outcomes with In-Utero Exposure to Indoor Macrocyclic Trichothecenes, *Stachybotrys*, and *Trichoderma* †

**DOI:** 10.3390/jof12070513

**Published:** 2026-07-13

**Authors:** Irene H. Grant, Noemi Olivo, Harriet Ammann

**Affiliations:** 1 Integrative Medicine Group, Tarrytown, NY 10591, USA; 2 Independent Researcher, Rochester, NY 14612, USA; olivo@mindspring.com; 3 Independent Researcher, Olympia, WA 98502, USA

**Keywords:** macrocyclic trichothecenes, *Stachybotrys*, Trichoderma, mycotoxin, indoor fungal contamination, indoor hazardous exposure, miscarriage, birth defects, biomarkers, lactation risk

## Abstract

**Background:** Produced by indoor *Stachybotrys* and *Trichoderma* spp., macrocyclic trichothecenes (MTs), a type of cytotoxic respirable molecule (<0.01–0.03 µm), inhibit protein/DNA/RNA production, damage mitochondria, and induce apoptosis. Dust-bound MTs remain toxic despite remediation/disinfection. Inhaled MTs easily cross tissue barriers, potentially reaching the placenta and the unborn. **Methods:** Retrospective medical record abstraction of pregnant females and offspring cohort exposed to indoor MTs, *Stachybotrys*, or *Trichoderma* to correlate professional indoor testing, exposure variables, mold species, environmental MTs, medical symptomatology, outcomes, and urine/milk MTs excretion. **Results:** In eight women from seven MT/mold-contaminated homes, with 21 pregnancies, complications occurred in 19 (90%) pregnancies, including miscarriages (38%) and premature labor (33%). Placental abnormalities in two women (25%) from the same home (calcification, chronic villitis, placental infarcts, double placenta, gritty membranitis). Birth defects in infants (38%) included renal hypertrophy, levocardia, patent foramen ovale, ventriculoseptal defect, ptosis, teeth, and “goosebump” black/grey skin discoloration. Later abnormalities included developmental delay, oropharyngeal hypotonic dysphagia, refractory eczema, and refractory perirectal rash progressing to intussusception. Lactation difficulties included grey-black oronasal drainage, thrush, projectile vomiting, choking, oropharyngeal neurologic damage, apnea, and respiratory arrest. *Aspergillus* +/− *Penicillium* exposure was documented in all, *Stachybotrys* (75%), *Chaetomium* (62%), *Trichoderma* (38%), and indoor MT contamination exposure (75%). **Conclusions:** In-utero indoor MTs and *Stachybotrys* exposure correlate strongly with adverse gestational, neonatal complications (miscarriage, congenital defects, and placental abnormalities). Exposure timing and severity correlate with adverse outcomes. Breastfeeding with indoor exposure appears hazardous. Environmental/human MTs testing appears useful for identifying MT contaminaion and/or exposure.

## 1. Introduction

Most pregnancies result in healthy babies, and most neonates thrive. Miscarriages occur more often in the first trimester, at a rate of 12–15%, with other serious complications of pregnancy in <10% of all pregnancies delivering live babies [1]. Unidentified causes are estimated to be 30–50% for miscarriages [1], as are the reasons for most cases of pre-eclampsia, intrauterine growth retardation, and premature labor. Exogenous exposures may be one contributing factor to these adverse events.

Adverse pregnancy outcomes linked to maternal mycotoxin exposure were reviewed in 2020 [2]. Of 17 studies in the literature, all addressed orally ingested mycotoxins, notably aflatoxin. Prenatal mold exposure in the home in Korea was linked to atopic dermatitis, and in China, prenatal exposure to deoxynivalenol (DON), a simple trichothecene, was linked to low birth weight [3,4,5,6]. Various mycotoxins were detected in amniotic fluid, probably mostly from food [7]. There are no reports of macrocyclic trichothecene exposure in pregnancy in humans. To our knowledge, apart from case reports, research into the adverse effects of indoor exposure to biocontamination with macrocyclic trichothecenes on pregnancy outcomes is lacking.

Macrocyclic trichothecenes (MTs), potent cytotoxic respirable molecules (<0.01–0.03 µm), inhibit intracellular protein/DNA/RNA production, damage mitochondria, and induce apoptosis [8,9,10,11,12,13,14,15,16]. Being both hydrophilic and lipophilic, MTs easily cross tissue barriers and penetrate cell membranes. Direct MT injury to the placentae and the unborn is plausible [15,16]. Examples of macrocyclic trichothecenes are Verrucarin A, Satratoxin G, Satratoxin H, and Roridin A. The LD50 dose of Verrucarin A is 1 mg/Kg body weight in mice, and those of Satratoxin G and Satratoxin H are 1.2 and 5.7 mg/Kg, respectively, all after intraperitoneal injection. In contrast, the LD50 of Ochratoxin A (not a trichothecene) is 29.4 mg/Kg [17,18].

Little data linking human illness to the MTs found in water-damaged homes exists since direct exposure studies are unethical. This study investigates correlations between environmental MTs and *Stachybotrys* and/or *Trichoderma* indoor exposure, indoor activities, and disease severity of obstetrical and neonatal outcomes.

Our objectives were to (1) define adverse obstetrical and neonatal outcomes linked to indoor MTs, *Stachybotrys*, or *Trichoderma* exposure, and (2) correlate exposure variables with adverse outcomes to identify hazardous exposure risks and markers.

## 2. Methods

This study is a focused epidemiological retrospective assessment of eight pregnant females with 21 conceptuses exposed in seven homes contaminated with indoor dust MTs and *Stachybotrys* contamination. Homes were assessed by professional environmental inspection, including professional contamination classification levels for water and sewage intrusion, temperature, humidity, visible microbial growth, fungal air spore trap testing [19], bulk sample fungal microscopy, ERMI analysis of mold-specific quantitative PCR [20], and dust MT measurement by ELISA (Realtime Laboratories, Lewisville, TX, USA) [21].

Retrospective medical record data abstraction designed for this study was used to anonymously correlate professional indoor classification and testing, exposure variables [date, duration, intensity, dust containment, dust-producing activities, personal protection], mold species, environmental MTs, medical symptomatology chronology, clinical examination findings, outcomes, and urine/milk MTs excretion.

Indoor contamination was ranked as follows:

Extreme for widespread water damage, >100 sq.ft. visible microbial growth, >50,000 airborne water damage indicator microfungal spores/m3 other than *Stachybotrys*, (*Alternaria*, *Aspergillus*, *Chaetomium*, *Cladosporium*, *Penicillium*), ≥30/m3 airborne *Stachybotrys* spores or prolific *Stachybotrys* growth on microscopy;

Severe for prolonged water intrusion damage, visible microbial growth >32 sq.ft., 10,000 airborne water-indicator microfungal spores/m3, or any airborne *Stachybotrys* spores; and

Moderate for prolonged humidity, ≥10 sq.ft. visible microbial growth, airborne water-indicator microfungal spores other than *Stahcybotrys* >10× outdoor control spore counts, or *Stachybotrys* growth on microscopy.

Mothers were clinically assessed by comprehensive medical intake and examination after pregnancy by one of the authors (IHG), usually on multiple occasions. Data on maternal and neonatal clinical outcomes were collected using a systematic and thorough data collection tool, to include the following: indoor contamination exposure data, including timing (frequency and duration) of exposures, estimated individual exposure severity (dust-producing activities, lack of personal exposure protection, and duration of exposure), timing of maternal pregnancy/postpartum and pediatric symptoms, signs, physical examinations and diagnoses. After miscarriage or childbirth, data on the loss of pregnancy or birth itself and the appearance of the placenta were collected from available hospital obstetric documentation. Medical data on each mother and child born to exposed mothers was extracted from medical and pediatric clinical notes, where available. Mothers were tested for the development of specific fungal quantitative IgG antibody responses in serum (Quest Diagnostics *Stachybotrys chartarum/atra* (RGm24) IgG by immunoassay and Hypersensitivity Pneumonitis Evaluation for *Trichoderma viride* by Double Diffusion and ImmunoCAP^®^ methodologies) and MT excretion in urine and breast milk measured by ELISA (Realtime Laboratories) [10].

Electronic extraction and analysis of longitudinal clinical, environmental, and laboratory data were conducted. Indoor exposure intensity, dust containment, personal protection, hazardous dust-producing indoor human activities, and building conditions were correlated with clinical outcomes. As all patients were referred for clinical review and provided all samples and data voluntarily to IHG, no additional ethical (IRB) review was warranted. All data is anonymized.

## 3. Results

Eight pregnant females and 21 in-utero conceptuses (eight miscarriages, 13 infants in 11 births) with exposure to seven *Stachybotrys*, *Trichoderma*, and/or MT-contaminated homes were retrospectively analyzed.

### 3.1. Environmental Analysis (Table 1)

#### 3.1.1. Demographics

Six of the seven homes were located in the Northeastern USA: two each in Pennsylvania and New York, and one each in New Jersey, Massachusetts, and Ohio. All were suburban residences, except for one urban house (Home #4) in Queens, New York.

**Table 1:** Maternal demographics, indoor exposure parameters, microfungi, and biomarkers.

**Mother**	**#1**	**#2**	**#3**	**#4**	**#5**	**#6**	**#7**	**#8**	**Total Mothers**	
**Home**	**#1**	**#2**	**#3**	**#4**	**#5**	**#6**	**#7**			
**County**	**Queens**	**Bucks**	**Union**	**Westchester**	**Norfolk**	**Alleghany**	**Cuyahoga**			
**State**	**NY**	**PA**	**NJ**	**NY**	**MA**	**PA**	**OH**	**OH**		
Professional Inspection	1	1	1	1	1	1	1	1	8	100%
Odor	1	1	1	1	1	1	1	1	8	100%
Visible Mold	1	1	1	1	1	1	1	1	8	100%
Uncontrolled Water Intrusion	1	1	1	1	1	1	1	1	8	100%
HVAC/Ducts Contamination	1	1	nd	nd	1	1	1	1	6	75%
Sewage Intrusion	0	1	1	0	1	0	0	0	3	38%
Air Spore Trap	1	1	1	1	1	1	1	1	7	88%
Swab Microscopy	1	1	1	1	1	1	1	1	8	100%
Dust ERMI PCR	1	0	1	0	1	1	1	1	6	75%
Dust Macrocyclic Trichothecenes	1	nd	nd	1	1	1	1	1	6	75%
Photos Extensive Water Damage	1	1	1	1	nd	1	1	1	7	88%
Condition 3 Active Growth	1	1	1	1	1	1	1	1	8	100%
Contamination Moderate	*								0	0%
Contamination Severe		1	1	1	1				5	62%
Contamination Extreme	1 *					1	1	1	3	38%
*Alternaria*	1		1		1	1	1	1	6	75%
*Aspergillus-Penicillium*	1	1	1	1	1	1	1	1	8	100%
*Aspergillus niger*			1		1	1	1	1	5	62%
*Chaetomium*	1		1		1		1	1	5	62%
*Cladosporium*			1	1	1	1	1	1	6	75%
*Fusarium*	0	0	0	0	0	0	0	0	0	0%
*Mucor-Rhizopus*	1		1		1	1	1	1	6	75%
*Penicillium*		1	1	1	1	1	1	1	7	88%
*Phoma*	0	0	0	0	0	0	0	0	0	0%
*Stachybotrys*	1	1	0	1	1	0	1	1	6	75%
*By Microscopy & Hyphae*	1	1	0	1	1	0	1	1	6	75%
*Airborne Stachybotrys*	0	1	0	1	0	0	0	0	2	25%
*Trichoderma*	0	0	1	0	0	0	1	1	3	38%
*Macrocyclic Trichothecenes—* *Maternal*	nd	1	1 Milk & Urine	1	nd	1	1	nd *	5	*62%*
*Macrocyclic Trichothecenes—* *Baby (urine)*	1	0	0	0	0	0	0	nd *	7	*88%*
*Maternal Stachybotrys IGG*	1	nd	1	1	N: d	1	1	nd *	5	*62%*

nd—not done. * At first Moderate, later Extreme. CONTAMINATION RANKING: Moderate: >10 sq.ft. visible growth; detectable *Stachybotrys* or *Chaetomium*; or 3 K–!0 K airborne counts of other microfungi or >10× outdoor counts. Severe: >32 sq.ft. visible growth; *Stachybotrys* proliferation on microscopy; airborne *Stachybotrys* or *Chaetomium*; or 10 K–50 K airborne spores of other microfungi. Extreme: Widespread >100 sq.ft. visible growth; >100/m3 airborne *Stachybotrys*; or >50 K/m3 airborne spores of other microfungi.

#### 3.1.2. Hazardous Indoor Conditions

All homes were professionally inspected and documented to be odorous, with visible microbial growth, uncontrolled high humidity, or uncontrolled water intrusion. Four of the seven homes (57%) had heating, ventilation, and air conditioning (HVAC) contamination, and three (43%) had sewage intrusion contamination. All seven homes were classified as Condition Three (active mold growth). Contamination classification ranged from Severe in five (62%) to Extreme in three (38%) homes.

As one example of the building issues, Home #7 was incorrectly constructed, promoting high humidity, water intrusion, and microbial contamination, including both *Stachybotrys* and *Trichoderma*. A professional inspection revealed widespread construction defects, including improper foundation drainage, missing window flashings, inappropriate vapor barrier placement preventing air circulation promoting humidity, and noncompliant backfill. Water intrusion, condensation, and interstitial moisture led to visible fungal growth, rusted fasteners, and damaged materials throughout the structure. Mold and environmental testing confirmed significant microbial contamination on all three floors, indicating grossly defective building practices and poor indoor air quality.

#### 3.1.3. Environmental Macrocyclic Trichothecenes

Macrocyclic trichothecenes (MTs) were detected in all five homes tested (Table 1). The two homes not tested for MTs were identified as highly probable for MT contamination based on airborne *Stachybotrys* spores with MTs detected in the mother’s urine (Home #2) and *Trichoderma* identified in dust and MTs in the mother’s urine and breast milk (Home #3).

#### 3.1.4. Environmental Microfungi

Air-spore trap testing and dust microscopy were done in all seven homes, and dust quantitative PCR analysis, as well as environmental dust mycotoxin testing, were done in five (71%).

*Stachybotrys chartarum* was found in six (75%) homes. Two homes had airborne *Stachybotrys* spores, and five homes (62%) had visible *Stachybotrys* growth detected by microscopy, with heavy proliferation in two. *Trichoderma* was found by dust PCR in two (29%) and microscopy in one (14%) home(s). Both *Stachybotrys* and *Trichoderma* spp. were present together in one (14%) extremely contaminated home (Home #7).

*Aspergillus* spp. was found in all homes, *Penicillium* spp. in six (86%), *Alternaria* and *Cladosporium* in five (71%), and *Chaetomium* spp. in four (57%). Other microfungi were present in these homes but were not included since they are not known to produce MTs, the focus of this report. Of note, *Fusarium*, the producer of simple trichothecenes such as DON, was not found in any of the homes.

### 3.2. Analysis of Maternal Exposures (Table 1, Table 2, Table 3 and Table 4)

#### 3.2.1. Maternal Exposure to Hazardous Conditions

Water damage and other hazardous conditions are shown in Table 1 and Table 4. All eight mothers were exposed to Condition 3 active mold growth, odors, visible mold growth, and uncontrolled water intrusion, with five (62%) exposed to HVAC contamination and three (38%) to sewage intrusion contamination. Three mothers (38%) were exposed to extreme contamination, and five (62%) were exposed to severe contamination.

#### 3.2.2. Maternal Exposure to Macrocyclic Trichothecenes (Table 2)

Six mothers were exposed to indoor dust MTs.

Two mothers from homes never tested for MTs were included based on exposure data, clinical symptomatology, Stachybotrys IGG, and MT results.

The pregnant woman in Home #2, which lacked MT testing, was exposed in the second trimester to airborne *Stachybotrys* and developed recurrent nosebleeds, severe headaches, abdominal pain, threatened miscarriage with unexplained vaginal bleeding, and detectable serum *Stachybotrys chartarum* IgG and MTs in her urine.

The mother from untested Home #3 was exposed to *Trichoderma* identified in dust and had a *Stachybotrys* IGG titer and MTs in her urine and breast milk. Home #3 was classified as extremely contaminated, with extensive, prolonged water intrusion and widespread visible mold growth, including *Trichoderma* contamination, but not *Stachybotrys*. However, the mycological testing was not thorough. The mother became severely ill, developing pneumonia in the second trimester. Her exposure stopped in the eighth month. Within a month postpartum, MTs were found in her breast milk, urine, and in the baby’s urine.

#### 3.2.3. Maternal Exposure to Indoor Microfungi (Table 2)

Maternal *Stachybotrys chartarum* exposure was found in six (75%) and 21% of conceptuses, and maternal *Trichoderma* exposure was found in 38% and 29% conceptuses. Two pregnant mothers were exposed to both *Stachybotrys* and *Trichoderma* spp. in an extremely contaminated home, Home #7. Maternal *Chaetomium* exposure was found in 62% and 43% of conceptuses.

All mothers were exposed to *Aspergillus* spp., with *Penicillium* spp. exposure in 88%, and *Alternaria* and *Cladosporium* exposure in 75%.

However, in Home #6, neither *Stachybotrys* nor *Trichoderma* was detected. Nevertheless, the mother in Home #6 had detectible serum *Stachybotrys* IgG and *Trichoderma* IgG titers. MTs were found in dust as well as in urine from the mother and all her children.

**Table 2:** Exposure to macrocyclic trichothecenes, *Stachybotrys*, and *Trichoderma.*

Exposure	Homes	Mothers	Conceptuses
	(*n* = 7)	(*n* = 8)	(*n* = 21)
Macrocyclic Trichothecenes *	5 (71%)	6 (75%0	Gestational Complications 19 (90%)
			(8 miscarriages, 11 births)
Stachybotrys **	5 (71%	6 (75%)	13 (21%
Trichoderma **	2 (29%)	3 (38%)	6 (29%)
*Chaetomium*	4 (57%)	5 (63%)	9 (43%)
*Aspergillus/Penicillium*	7 (100%)	8 (100%)	21 (100%)

I* Only five homes tested. ** Produces macrocyclic trichothecenes.

#### 3.2.4. Risks and Timing of Maternal Exposures (Table 3)

Six mothers (75%) were exposed to uncontained demolition, mold disturbance, and personal unprotected exposure while cleaning. Five (63%) were exposed to HVAC or basement contamination.

**Table 3:** Maternal indoor exposure risks in homes ranked by worst intrapartum and pediatric outcomes.

Mothers	Home	Successful Birth Before Exposure	Exposure Duration Before Conception	Miscarriage During Exposure	Exposure End	Successful Birth After Exposure End	Demolition	Intense Personal Unprotected Exposure	Mold Disturbed Without Containment	HVAC or AC Contamination	Basement Exposure
#1	Home #1	1	INTRAPARTUM: [3rd trimester 7th mo.]	0	1 year	nd	1	0	1	1	0
#2	Home #2	1	INTRAPARTUM: [2nd trimester 4th mo.]	Vaginal bleeding	4 mo.	nd	0	1	0	1	0
#8 *	Home #7	0	INTRAPARTUM: [3rd trimester 6th mo.]	Preterm Labor	4 mo.	1	1	1	1	1	1
#3	Home #3	1	INTRAPARTUM: [1st trimester 3d mo.]	0	5 mo.	nd	0	1	0	0	1
#4	Home #4	1	1 year	2	Continued	nd	1	0	1	0	1
#5	Home #5	1	INTRAPARTUM: [2nd trimester]	1	Continued	nd	1	1	1	1	1
#6	Home #6	1	Several months	4	3 years	nd	1	1	1	0	
#7	Home #7	1	INTRAPARTUM: [1st trimester 3d mo.]	1	5 years	1	1	1	1	1	1
TOTAL Mothers 8	TOTAL Homes 7	Prior births 7	Pregnant at occupancy 6	Miscarriages 8		2	6	6	6	5	5
%							*75%*	*75%*	*75%*	*63%*	*63%*

nd—not done. * Sister of mother from Home #7.

Seven (99%) mothers had had prior successful births before exposure. Six mothers were pregnant at the time of occupancy: two in the third trimester, two in the second trimester, and two in the first trimester. The earlier in the pregnancy, the worse the outcome. All miscarriages occurred within the first one to two years of occupancy and during occupancy (Table 4 and Table 5). All four of the mothers who miscarried were exposed to demolition and uncontained mold disturbance, with three being intensely exposed without personal protection. The pregnant mother from Home #2 developed abdominal pain and bleeding in the second trimester, consistent with threatened miscarriage.

**Table 4:** Ranking methodology for hazardous environmental exposure based on in-utero and pediatric outcome severity.

**RANKING METHODOLOGY:**		
CLINICAL OUTCOME		1 Moderate, 2 Severe, 3 Extreme [hospitalization], 4 Death
INDOOR MOLD CONTAMINATION SCORE:	Moderate	Visible growth 10 sq feet Any detectible of *Stachybotrys* or *Chaetomium* >10× airborne spore counts of water damage indicator microfungi [e.g., *Alternaria*, *Aspergillus*, *Chaetomium*, *Cladosporium*, *Memmoniella*, *Penicillium*, *Trichoderma*] vs. outdoors 3000–10,000 airborne spores of other indoor microfungi > 10× outdoor counts {e.g., *Aspergillus*, *Penicillium*]
	Severe	>32 sq.ft visible microbial growth Airborne *Stachybotrys* or *Chaetomium* Active *Stachybotrys* proliferation on specimens by microscopy 10,000–50,000/m^3^ airborne spores of other indoor water-indicator microfungi
	Extreme	>100 sq feet visible microbial growth widespread
		*Stachybotrys* airborne spore counts > 30/m3
		>50,000/m3 airborne spores of other indoor water-indicator microfungi Heavy *Stachybotrys* proliferation on specimens by microscopy
INDOOR WATER SCORE	Moderate	Prolonged humidity, chronic leaking, with scattered visible damage
	Severe	Prolonged water intrusion or stagnation for >96 h with extensive visible damage
	Extreme	Unabated, continuous intrusion flooding for weeks
INDOOR STACHYBOTRYS CONTAMINATION:	Moderate	Any detection; Severe: Airborne spores or microscopy prolific growth
	Severe	Airborne > 10/m3
	Extreme	Airborne > 30/m3
		Heavy proliferation by microscopy

All eight mothers moved out, and five (63%) became homeless, looking for a new residence. Four (50%) lost most of their belongings. Four (50%) remained ill despite discontinuing exposure.

Mothers who continued to be re-exposed over the years became progressively chronically ill. In contrast, the two mothers exposed to an extremely contaminated home, Home #7, suffering multiple gestational complications and sick children, both went on to have uncomplicated, successful births later on.

**Table 5:** Timing of exposures, testing, and outcomes.

	Exposure Onset	Exposure Timing to Pregnancy	Time from Acute Exposure to Envir. Testing	Date Dust MT Test	Outcome	Date Med. Evaluation	Date Maternal MT Test	Expo. to Eval. Time *	Comments
#1	Aug. 2013 occupied musty home with visible water leaks and mold in vents. Jun. 2014 heavy rain leaks. Aug. 2014. construction repairs.	Third trimester, seventh mo. Birth Nov.’2013. Continuous lactation until Sep. 2015.	Four months: Aug. 2013–Sep. 2014 Environment *Stachybotrys* *Chaetomium* MTs	Feb. 2015	Nov. 2013 birth. Aug. 2014 Extreme Exposure. Hospitalized in Sep. 2014. Intussusception.	Nov. 2014	Dec. 2014	2 mos.	Chronic roof leaking. Mother moved in second trimester, promptly developed gestational diabetes, rhinorrhea, and cough. After birth, baby developed rashes, grey tongue, and refractory diaper rash. After uncontained repairs in Aug. 2014, whole family ill with respiratory symptoms. Baby developed grey-black tongue/nasal discharge, bleeding blackish perianal rash, hospitalized with intussusception.
#2	Jun. 2014 occupied musty home with visible water leaks and mold in vents.	Second and third trimester. Moved out in eighth month.	Four months: During third trimester *Stachybotrys*	nd	Moved out before delivery. Baby did not tolerate breast milk.	Sep. 2015	Jun. 2015	11 mos.	Mother moved in second trimester, promptly ill with vomiting, diarrhea, rashes, chronic sinusitis, vaginal bleeding, and persistent monocytosis. Airborne *Stachybotrys* found in third trimester, and she moved out before normal delivery. Baby did not tolerate breast milk.
#3	Dec. 2011 occupied musty home with visible mold and continuous water pipe leak.	First trimester Dec. 2011–Jun. 2012. Jun. 2012, moved out in third trimester, ninth mo. Jul. 1, 2012, Birth.	Eight months: Dec.’11–Jun. 2012 *Chaetomium*, *Trichoderma*	nd	Congenital heart VSD, oropharyngeal paresis after MT+ breastmilk Ingestion for two weeks.	Nov. 2012	Jul. 2012 Urine and Milk	within 6 wks.	Mother with prior pregnancy with gestational diabetes and preterm labor. After moving in, mother and 1.3-year-old son had progressive respiratory symptoms. She developed pneumonia in the second trimester. Baby had heart defect, developed blackened oronasal drainage and neurological swallowing impairment after MT+ breastmilk ingestion.
#4	1993 occupied old, musty house: prompt onset chronic sinusitis and headaches.	Conceived within one year of moving in and miscarried twice, in 1994 and 1997.	15 years: 2007 uncontained remediation triggered illness. 2008 found widespread growth and airborne *Stachybotrys*	2007	Two miscarriages, 1994 and 1997, within 3 years after occupancy.	2008	2007	15 yrs.	Normal birth two years before moving into old house, then had two miscarriages. After acute xposure illness in 2007 from uncontained remediation, found airborne *Stachybotrys* in basement and MTs in dust and urine.
#5	May 2005 occupied musty house with recurrent septic overflow.	Second trimester moved in: normal birth. 2007 First-trimester miscarriage, 2008 IUGR, premature birth.	10 years: 2005 -Nov. 2015: VAC and Refrigerator coil dust MT+, *Stachybotrys* *Chaetomium*	Nov. 2015	2005 normal birth; 2007 miscarriage; 2008 IUGR, birth of Premature, stiff, diffusely red baby.	Oct. 2015	Jun. 2015	10 yrs.	Mother moved in 2005 in the second trimester, promptly developed sinusitis, later requiring sinus surgery twice. Birth normal. First-trimester miscarriage in 2007. Intrauterine growth retardation [IUGR] with preterm labor in 2008 and stiff, diffusely red baby.
#6	Mar. 2011 occupied. Apr. 2012 chronic leak. May 2014 faulty repair increased water intrusion. Aug. 2014 visible mold.	Third trimester moved in: normal birth. 2011–2012 Four miscarriages. Apr. 2012 Leak discovered. Sep. 2012 Conceived: twins born May 2013.	Three years: Apr. 2012 Leak found after torrential rain. Chronic basement intrusion. Jun. 2014–Dec. 15 Severe progressive uncontrolled intrusion. HVAC and refrigerator coil dust MTs+	Dec. 2015	Four miscarriages. Sep. 2012 twins conceived and born prematurely May 2013.	Feb. 2016	Jun. 2015	2 yrs.	One twin with strabismus, the other developmental delay, decreased fine motor, and foot drag ataxia. Both developed upper respiratory symptoms, cough, and skin blisters. Family ill with diarrhea. Moved out Apr. 2015.
#7	Feb. 2008 occupied home with poor ventilation, excess humidity, and recurrent repairs. 2010 construction and remodeling.	2008–2012 Five pregnancies. 2009 First-trimester miscarriage.	Six years: Feb. 2008–Dec. 2013 *Stachybotrys* *Trichoderma* *Chaetomium* MTs	Jun. 2014	One miscarriage, multiple pregnancies, ill children.	Jun. 2014	Jun. 2014	2 yrs.	Multiple pregnancies with complications including miscarriage, hospitalizations, congenital anomalies, abnormal placentas corresponding to timing of home construction and remodeling [See Section 3.5. Placental Abnormalities and Section 3.8.1.B. Epidemiological Clustering of Abnormal Placentas and Congenital Defects After Uncontained Basement Construction and Remodeling].
#8	May 2011–Jul. 2011 occupied after recent remodeling of Home #7.	May–Jul. 2011, Second trimester (six mo.). Jul. 2011 Preterm C-Section 36 wks.	Three months: May–Jul. 2011 *Stachybotrys* *Trichoderma* *Chaetomium* MTs	Jun. 2014	Eclampsia. Baby premature with black-grey coarse skin, breastmilk intolerance. Abnormal placenta.	Feb. 2017	Could not afford	6 yrs.	Developed Eclampsia and chronic sinusitis after moving in. Baby premature with abnormal discolored black-grey placenta and skin, and breastmilk intolerance with choking requiring emergency room care.

* Time between last exposure and medical evaluation. Mother from Home #8’s first pregnancy; all other mothers had one prior pregnancy. nd not done.

#### 3.2.5. Matching Maternal Symptom Progression with Visible Mold Contamination (Table 4 and Table 5)

Home #6 had chronic recurrent unabated water intrusions over a year from torrential rain flooding into the basement, eventually dislodging the foundation of the house. While MTs were found in this home, neither *Stachybotrys* nor *Trichoderma* was detected. The mother moved in eight months pregnant, delivering with a normal birth, followed by four miscarriages, before conceiving twins later born prematurely. Faulty construction resulted in continuous water intrusion, and visible mold appeared in the basement. The mother developed blistering rashes and vaginal pain, while her firstborn daughter had burning dysuria.

After the whole family developed burning diarrhea, they moved out. The mother continued with chronic persistent pain in her neck, shoulders, legs, vagina, and bladder. Re-entry into the home triggered red eyes, nasal congestion, throat swelling, bladder pain, rectal fissures, rectal bleeding, burning sensations (scalp, eyes, bladder, and urine), hoarseness, loss of voice, cough, cognitive impairment (impaired memory, forgetfulness, getting lost), and twitches and jerks. On examination, the mother had scleritis, diffuse mucosal inflammation with ulcerative nasal mucositis, oral thrush, soft palate inflammation, pharyngeal cobblestone lymphatic hyperplasia, and tender lymphadenopathy in the neck and groin, and a left lower lobe lung nodule on imaging. Urinalysis showed leukocytes without bacteria on culture. Both *Stachybotrys* IgG and *Trichoderma* IgG were detected. Urine MTs were elevated in the mother and all of her children.

Her firstborn daughter developed chronic nausea, anorexia, vomiting, and dysuria. Her second-born twin son, a toddler, developed heavy nosebleeds, a chronic cough, developmental delay, foot drop, decreased fine motor skills, and blistering rash. She and the twin girl toddler had strabismus and developed a chronic cough and oral thrush.

On follow-up a year later, after moving out, symptoms resolved, and MTs were no longer detected in their urine.

### 3.3. Maternal Clinical Observations

Six (75%) mothers were White, and two were Hispanic (Home #7). All mothers, except for the mother in Home #8, had prior uncomplicated pregnancies, birthing healthy children.

Six (75%) gravidas experienced unexplained severe fatigue, skin symptoms (four rashes, three redness, four bruising), nasal symptoms (six nasal congestion, five postnasal drainage, four nostril drainage, four sinus congestion, four sinus pain, and three sinusitis), throat symptoms (six chronic hoarseness or loss of voice, four chronic throat pain), and respiratory symptoms (six chronic cough, three shortness of breath).

Five (63%) had headaches, eye irritation, ear symptoms (five tinnitus, three ear pain), nasal congestion, sinus pain, and GI symptoms (five nausea or diarrhea, three fluctuating irritable bowel symptoms).

Neurological symptoms were common: six (75%) mothers had impaired concentration, memory, focus, and/or attention; four (50%) had twitches, uncontrolled jerks, agitation, and anxiety and/or depression; three (38%) had signs of encephalopathy, delirium, agitation, aphasia, disorganization, and/or vertigo.

Five (63%) experienced periorbital edema, cervical-submandibular lymphadenopathy (four neck). All five of the mothers who developed sinusitis while pregnant were MTs-exposed.

Four (50%) experienced unexplained body burning sensations, including burning skin and urine with normal urinalysis, severe unexplained refractory pain, multiple chemical intolerance “sensitivity”, facial pain, unexplained bruising, postnasal drainage, sinus pain, chronic pharyngitis, and muscle pain.

Upon physical examination, throat inflammation with injected capillaries and lymphoid hyperplasia was found in all eight mothers (100%), nasal findings (red raw nostril mucosa in 7/8 (88%), red turbinates 5/8 (63%), loss of nasal mucosa and hairs in 4/8 (50%)), palpable cervical submandibular lymph nodes in 5/8 (63%), tonsillar enlargement in 6/8 (75%), and oral pathology in 6/9 (67%), including abnormally swollen soft palette and swollen deformed uvula.

Of the five mothers tested, *Stachybotrys* IgG was detected, but was not elevated. Of the 3 mothers not tested for *Stachybotrys* IgG testing, all were exposed to severe indoor *Stachybotrys* contamination. Of the three mothers tested for *Trichoderma* IgG, low levels were detected in two. MTs were found in the urine of all five mothers tested (62%) and in the breast milk and urine of one, as well as her baby’s urine. Of the three mothers not tested for MTs, all were exposed to indoor MTs and heavy indoor *Stachybotrys* growth, and one untested mother’s baby had detectable urine MTs.

### 3.4. Pregnancy Complications (Table 6)

Seven mothers (88%) lacked underlying health problems before this exposure. The mother from Home #3 (with the MT+ breast milk and urine) had pre-existing conditions, including Factor V Leiden mutation, polycystic ovary syndrome, gestational diabetes with her first child, and chronic fatigue syndrome before this pregnancy.

**Table 6:** Clinical maternal/child outcomes and macrocyclic trichothecenes/mold exposure.

Clinical Outcomes	Number	Indoor MT-Exposed	*Stachybotrys*-Exposed	*Trichoderma*- Exposed	Comments
Mothers	8	6	6	3	4/8 (50%) mothers received MT testing (4 Urine, 1 breast milk)
Total Pregnancies	21	19	19	7	15 First Trimester Exposures
					4 Second Trimester
					2 Third Trimester
Gestational Complications	19/21 (90%)	8 Miscarriages 11 Births	8 Miscarriages 11 Births	[6/7 also exposed to Stachybotrys]	8 Miscarriages 5 Preterm Labor 1 Pre-eclampsia
Miscarriages	8 (38%)	8 (100%)	4 (50%)	0	
Births	13	11	9	3	6/13 (46%) babies had urine MT testing
	7 Male, 6 Female				
Placental Abnormalities	3 (100%)	3 (100%)	3 (100%)	3 (100%)	Calcification Chronic villitis with placental infarcts Double placenta Gritty membranitis
Birth Defects	5	4	4	3	2 Cardiac (PDA, VSD) 2 renal hypertrophies 1 ptosis
Neonatal Dermatologic Complications	6	4	4	3	1 Severe refractory eczema 1 bleeding refractory perirectal “diaper rash” that later progressed to intussusception 1 diffuse permanent “goosebump” textured black-grey skin mottling
Neonatal Neurologic Complications	6	4	4	3	6 Developmental delays 1 ptosis 1 oropharyngeal hypotonic dysphagia

Six (75%) mothers had 19 (90%) gestational complications, with eight miscarriages and 13 babies: eight miscarriages and five congenital anomalies (Table 3, Table 4, Table 5 and Table 6). There were eight (38%) first-trimester miscarriages, all in mothers exposed to MTs, and 4/8 (50%) exposed to *Stachybotrys*. All miscarriages occurred while the mothers were occupying the contaminated residence. Three gravidas were exposed to high levels of *Stachybotrys*; two of them were also exposed to *Chaetomium*, and one to *Trichoderma*. All moved out.

The mother from Home #2 developed vaginal bleeding, nausea, vomiting, and abdominal pain with exposure to airborne *Stachybotrys* in the second trimester.

Seven other pregnancy complications occurred in MT-exposed mothers: seven with premature labor, two intrauterine growth retardation, one severe preeclampsia, and two fetal renal hypertrophies.

### 3.5. Placental Abnormalities

Three abnormal placentas were documented from two gravidas (from Homes #7 and #8) exposed to Home #7 (see Section 3.8.1.B for detailed chronology).

Mother #7 from Home #7 had two pregnancies where the placentae were analyzed at pathology. The first placenta, collected six months after she moved in, showed calcifications. Her second placenta, collected two years after exposure to uncontained construction and remodeling, was strikingly abnormal: a gritty, double placenta with membranitis (inflammation of the membrane on the outer surface of the placenta on the infant’s side). The maternal surface was diffusely covered with firm, salt-like granules and loosely adherent dark brown, blood-clot sponge-like tissue with granular material. No diagnostic studies were done. At birth, the baby’s skin was strikingly red and was later diagnosed as severe eczema refractory to steroids.

The mother from Home #8, the six-month-pregnant sister of the mother from Home #7, moved into Home #7 less than six months after the uncontained remodeling construction was completed. She developed preterm labor and preeclampsia with emergency C-section for a weak fetal heart rate. This third placenta was unexpectedly small for gestational age, with tan-grey membranes, small chronic infarcts, and dark grey discoloration on the fetal side, with dark grey vessels radiating towards the umbilical cord insertion, and yellow lesions on the maternal side, diagnosed on pathology as “chronic focal villitis (inflammation) of unknown etiology”. Her baby’s skin was also unusual, with a sandpaper texture and permanent dark grey mottled discoloration, unlike his parents’ skin.

### 3.6. Birth Defects (Table 6)

Birth defects occurred in 8/13 (40%) neonates, of whom five (63%) were MT-exposed. Enlarged kidneys were seen in two, as documented by ultrasound prior to birth. Two babies had cardiac defects (levocardia with patent foramen ovale and one ventriculoseptal defect). One baby had two fully developed teeth. Six neonates had unusual skin changes: two with diffuse redness. The Hispanic baby of the mother from Home #8 was born with unusual widespread mottled blotchy permanent sandpapery “goosebump” melanotic grey skin discoloration, unlike his parents’ skin. The placenta also had dark grey discoloration.

### 3.7. Breastfeeding Complications (Table 7)

Adverse lactation consequences occurred in the majority that were breastfed, 7/11 (64%): three grey-black oronasal drainage, four projectile vomiting, three choking, two oronasal bleeding, two grey-black tongue coat, one oropharyngeal neurologic damage, one apnea/respiratory arrest, one intussusception, and one refractory perirectal rash. Of the six babies with unusual rashes, including two with diffuse unexplained redness, four were breastfed, and two either did not tolerate or refused breastfeeding.

No significant complications occurred in the two babies who were never breastfed.

The baby from Home #3 was exposed in the first trimester and born with a VSD. In the first two weeks, he failed to gain weight, despite frequent feeding. By the third week, breastfeeding triggered excessive spitting, coughing, crying, projectile vomiting, choking, and brown-black nasal drainage. Macrocyclic trichothecenes were detected in the mother’s breast milk and urine. At four weeks, when breastfeeding was discontinued, the nasal drainage stopped. Two weeks later, the baby was found developmentally delayed. With fluoroscopy, a pediatric neurologist diagnosed the infant’s inability to suck or swallow as oropharyngeal dysphagia, and neuromuscular paresis with due to neurotoxicity from MT ingestion.

The baby from Home #1 was breastfed for months without serious symptoms other than chronic cough, persistent diaper rash, and loose stools, despite visible mold in the vents. However, eight months later, after acute unprotected exposure to uncontained mold disturbing roof and ventilation repairs of ducts heavily contaminated with *Stachybotrys* and MTs, the baby acutely developed black-gray thrush, breastmilk intolerance, bleeding rectal rash, near rectal prolapse, elevated urine MTs, and hospitalization for intestinal intussusception (see Section 3.8.1.A).

The baby from Home #2, with elevated airborne *Stachybotrys* spores, was exposed between the second and third trimesters, moving out in the eighth month before birth. This baby was unable to tolerate breastfeeding, experienced developmental delay, and also developed rashes.

Multiple babies exposed to Home #7 developed complications with breastfeeding (see Section 3.8.1.B).

**Table 7:** Breastfeeding complications.

Lactation Complications	Number of Children Born *n* = 13	Comments
Lactation Exposure	10/13 (76%)	
Milk Intolerance	7/10 (70%)	
Projectile Vomiting	3/10 (30%)	2 Hospitalized
Choking	3/10 (30%)	1 Oropharyngeal Paresis from MT+ Breast Milk
		2 Environmental MTs
Grey/Black Tongue *	3	1 MT+ Breast Milk/
		2 Environmental MTs
Oronasal Bleeding	2	2 Environmental MTs
Refractory Bleeding Perirectal “Diaper Rash” *	1	Grey/Black Tongue
Intussusception *	1	Grey/Black Tongue
Apnea/Pulmonary Hemorrhage/ICU	1	Choking
Oropharyngeal Motor Paresis after MT+ Breast Milk ingestion	1	Grey/Black Tongue

* Same infant.

### 3.8. Determining Exposure Hazards Based on Pediatric Outcomes (Table 6)

Ranking indoor environmental contamination variables by severity of pediatric outcomes showed that the worst outcomes, miscarriages, paralleled the severity of water intrusion. Severe outcomes also paralleled indoor MT, *Stachybotrys*, and *Trichoderma* contamination. All miscarriages occurred in MT contaminated homes. *Stachybotrys* was found in 5/7 homes (71%). However, while neither *Stachybotrys* nor *Trichoderma* was found in Home #6 on environmental testing, the mother from Home #6, who had four miscarriages, had IgG titers to both *Stachybotrys* and *Trichoderma* and MTs in her urine. Urine MTs were also found in all three of her children.

#### 3.8.1. Adverse Maternal/Pediatric Outcomes Temporally Related to Uncontained Construction Repairs

In two homes, uncontained construction clearly triggered pediatric hospitalizations.

##### 3.8.1.A. Repairs Triggered Breastmilk Intolerance, Grey Discoloration of Togue, Nasal Discharge, Rectal Bleeding, and Progressed to Intussusception

The mother from Home #1 developed gestational DM while seven months pregnant after moving into Home #1 with visible mold contamination in the air vents. The baby was born with two fully developed teeth. Sleeping and breastfeeding under the leaking, visibly contaminated, blackened air vent, the infant promptly developed chronic cough, loose stools, and a refractory anal “diaper” rash. Six months later, after recurrent roof leaking from torrential rain and turning on the AC, the whole family became ill. Within 3–4 weeks after roof and vent repairs were done without containment (i.e., air separation from the inside of the home), the baby became progressively ill with a grey-black tongue coat and nasal discharge, noisy breathing “snuffles”, stridor, cough, vomiting, rectal rash with bleeding, followed by hospitalization for near rectal prolapse and intussusception. Microscopy of both sheet rock and HVAC dust showed prolific *Stachybotrys* growth and MTs.

##### 3.8.1.B. Epidemiological Clustering of Abnormal Placentas and Congenital Defects After Uncontained Basement Construction and Remodeling

Home #7 was poorly constructed with inadequate air circulation, excessive indoor humidity, and extreme mold (*Stachybotrys*, *Trichoderma*, and *Chaetomium*) and MT contamination.

In February 2008, a three-month-pregnant mother moved into Home #7 with stagnant yard water. She routinely cleaned the house herself without protection. In August 2008, at birth, her second baby’s placenta showed unexplained inflammation and calcification. In January 2009, dilatation and curettage were done for a nonviable embryo. In November 2009, her third baby was born with swollen kidneys.

Between June and September 2010, during the first trimester of her fourth baby, the basement was remodeled without containment.

In January 2011, a baby boy was born with congenital heart disease (levocardia with patent foramen ovale). The placenta was not examined. Breastfeeding stimulated projectile vomiting and choking. His breathing stopped, requiring weeks of hospital intensive care for pulmonary hemorrhage.

In May 2011, less than a year after the remodeling construction, the six-month-pregnant mother #8, the sister of mother #7, moved into Home #7 and developed preeclampsia in July 2011, requiring a C-section due to weak fetal heart function, delivering a 36-week premature boy. The placenta had small chronic infarcts, dark grey discoloration on the fetal side, and yellow lesions on the maternal side, diagnosed as “chronic villitis (inflammation) of unknown etiology “on pathology. Her baby’s skin was also unusual, with a sandpaper texture and permanent dark grey mottled discoloration, unlike his parents’ skin (see Section 3.5).

After returning to Home #7, the breastfed baby also developed choking, requiring urgent hospital care.

In January 2012, 1.5 years after the basement remodeling, the mother #7 delivered her fifth baby. This placenta was also abnormal: a gritty, double placenta with membranitis (inflammation of the membrane on the outer surface of the placenta on the baby’s side). The maternal surface was diffusely covered with firm salt-like granules and loosely adherent dark brown blood clot sponge-like tissue. No other diagnostic studies were done. Breastfeeding was tolerated, but the baby developed a diffuse, refractory chronic dermatitis.

In September 2013, three years after the remodeling, mother #7’s sixth baby was born without complication, was never breastfed, and remained healthy.

## 4. Discussion

### 4.1. Principal Findings

This real-world observational retrospective analysis supports a significant correlation between exposure of pregnant or nursing mothers and their newborns to indoor MTs, *Stachybotrys*, or *Trichoderma* and serious, even life-threatening medical outcomes, especially for the unborn and neonate. At present, there is no epidemiological evidence directly linking macrocyclic trichothecenes, *Stachybotrys*, or *Trichoderma* exposure with obstetrical or neonatal complications. A major limitation in this topic area is the lack of quality human data for this underrecognized exposure. Since controlled clinical trials to investigate such exposures in any humans, especially pregnant women and neonates, would be unethical, detailed observational and environmental studies are essential for advancing knowledge.

In this study, severe pregnancy outcomes correlated with exposure to dust-dispersing, air-contaminating activities as well as unprotected personal exposure. The worst outcomes were from exposures to uncontained construction, demolition, remodeling, and contaminated ventilation systems. Indoor sewage contamination appears particularly hazardous. Heavy water intrusion, the main risk for *Stachybotrys* proliferation, was also the most frequent environmental variable associated with significant illness. Accurately documenting exposure severity and duration is inherently challenging in a study of this type, but water intrusion was key in all cases.

With the most common symptomatology being respiratory and neurological, inhalation was presumed to be the most frequent route of exposure in the mother’s homes, leading to illness. Dermal contact and ingestion symptoms were less frequent. Two important caveats remain, however: (1) other environmental toxins, such as mycotoxins or volatile organic compounds, could be fully or partly responsible for the disease observed, and (2) the diagnostic accuracy of the MT assay(s) is not documented, and its validation is weak.

### 4.2. Results in the Context of What Is Known

The maternal symptoms and physical findings described here correspond with military medicine reports of MT injury to skin and respiratory or gastrointestinal mucosa [8,16]. Inhalation of MTs appears to be the most toxic in prior reports. Maternal burning and bleeding were frequent symptoms, with oronasal pharyngeal mucosal barrier defense injuries the most common sites involved, consistent with both animal model and military data [8,21]. Neurological symptomatology, including maternal cognitive and pediatric neurodevelopmental impairment, also matched animal research showing inhaled trichothecenes injure mucosa on contact and rapidly spread directly into the brain from the nasal mucosa via olfactory cranial nerve axonal penetration [22].

Being amphiphilic molecules (both polar hydrophilic and lipophilic), MTs easily enter the body in direct contact with any tissue. Inhalation is the most efficient and harmful route of exposure [8,16,23,24,25,26]. As respirable molecules (<0.01–0.03 microns), they reach deeply into the lung periphery, inducing alveolar macrophage apoptosis, and penetrate directly into the systemic circulatory system [11,16,24,25,27]. MTs form covalent protein adducts, causing multiple toxic pathophysiological effects dependent on dose and site reached, through tight covalent binding to serum and tissue macromolecules [14].

In water-damaged indoor environments, macrocyclic trichothecenes accumulate in large quantities in *Stachybotrys* biofilm fragments and debris, as well as dust [28]. Potentially a serious, persistent indoor health hazard, they remain toxic for prolonged duration, if not indefinitely, despite remediation and disinfection by standard methods, including heat, since they are heat-stable [8,16,27,29,30]. While MTs are not volatile themselves, they attach to minuscule, highly respirable particulates (0.03–0.3 microns), and accumulate in indoor dust, especially from cellulose-containing building materials, such as straw, fiberboard, gypsum, wallboard, ceiling tile, wallpaper, lint, and belongings [9,31,32,33,34,35]. Activities disturbing dust in *Stachybotrys*-contaminated homes increase airborne MTs and the risk of MT inhalation and ingestion through indoor food contamination [29,36].

MTs are not produced by microfungi found in the food chain, such as *Fusarium*. *Stachybotrys* is a marker for indoor water intrusion, and its MTs are potential unique markers for indoor contamination.

Mechanisms of MT toxicity are multiple, including cytotoxicity and apoptosis via oxidative stress, protein synthesis inhibition, inflammatory gene expression via ribotoxic stress response involving activation of P38 mitogen-activated protein kinases (MA PKs)/JNK, RNA/DNA synthesis inhibition, opening phosphorescent pt (II)-coproporphyrin, and mitochondrial damage through loss of transmembrane potential and mitochondrial translation inhibition [8,9,10,12,13,14,15,16]. MTs block protein synthesis processes by inactivating ribosomal peptidyl transferase [8,10,11,14,15,16,37]. They also induce cellular apoptosis [10,14] and can trigger inflammatory cytokine cascades [13,38]. They impair mucosal barriers in the airways and gut, sometimes leading to ulceration, with epistaxis being the most frequent [8,16,28,29,31,39,40]. The potency of different MTs varies with Type D MTs 10 to 100 times more potent than type A or type D in activating MAPKs, impairing leukocyte proliferation, and inducing apoptosis [14,37].

Diagnosing MT exposure requires epidemiologically linking exposure to environmental *Stachybotrys* and/or MT contamination with consistent clinical symptomatology and biomarkers, such as urine or tissue MTs [27,28,33]. MTs have been detected in multiple human specimens from people exposed to water-damaged *Stachybotrys*-contaminated environments [21,23,29].

Neonatal outcomes reported here correspond in part to the route of exposure: (1) cutaneous (rashes, blistering); (2) inhalation (both neurological developmental delays and respiratory symptoms); and (3) ingestion (oropharyngeal paresis, diarrhea, rectal bleeding, prolapse, and intussusception). Other correlations may have been missed due to difficulty evaluating mucosal injury in children.

Neonatal breast milk intolerance occurred in 7/10 (70%). In one baby, ingestion of MT-contaminated milk caused fluoroscopy-confirmed oropharyngeal motor nerve paresis. Another baby progressed to respiratory arrest and pulmonary hemorrhage requiring ICU care. Thrasher et al. also described a 16-month-old infant who died with upper airway bleeding, pulmonary hemorrhage after exposure to a water-damaged home contaminated with *Stachybotrys* and MTs. At autopsy, trichothecenes were found in his lung, liver, and brain [40].

Most research on pregnancy exposure has focused on the simple trichothecenes of *Fusarium* contaminating food and grains, such as Type A T-2 toxin, and Type B Deoxynivalenol (DON) and nivalenol. DON crosses the placenta in sows [41] and causes reproductive toxicity and fetal abnormalities in experimental animals [42]. Intravenous T2 toxin-induced abortion and swine, but placentas were not evaluated [43]. In a dual-perfusion ex vivo model with five-term human placentae, DON transferred across the placenta, with approximately 20% of the maternal concentration detected on the fetal side [40]. In in-vitro placenta and in human biomonitoring studies, DON was found in serum and blood of pregnant women [44] and has been linked with autism in children [45]. DON and nivalenol were also found in first-trimester human amniotic fluid sampling in a study screening pregnancies for genetic risks [7].

Our high percentage of miscarriages and congenital anomalies (CAs) in a small group of women with similar indoor exposures is striking. Trichothecenes are known teratogenic and mutagenic chemicals impairing protein and DNA synthesis, causing cellular apoptosis. In animal studies, Trichothecenes cause both miscarriage and congenital anomalies. In humans, congenital anomalies are one of the main causes of fetal death, infant mortality, and morbidity. The prevalence of congenital anomalies in Europe between 2003 and 2007 was estimated to be 23.9 per 1000 births, and the associated perinatal mortality was approximately 9.2 per 10,000 births [46]. According to the World Health Organization, each year, 3.2 million children worldwide are born with Cas, and approximately 300,000 newborns with a diagnosis of birth defect die within the first 28 days [47]. In the United States, CAs account for 15 to 30% of pediatric hospitalizations. Approximately 25% of infant mortality is due to CAs [47].

Recently, the importance of environmental factors such as chemicals, infections, and maternal socioeconomic demographic factors has been emphasized, including prenatal air pollution exposure [46,47]. In many cases, the cause is unknown and hypothesized to develop during the first trimester as a result of multifactorial genetic interactions with environmental factors: preconceptional mutagenic, postconceptual teratogenic, and periconceptional endocrine disruption or epigenetic [47].

Spontaneous miscarriages are estimated to occur in about 20–25% of pregnancies in the United States [48,49,50]. Similarly, the causes of miscarriages are largely unknown. While environmental factors and genetic predisposition are suspected, few studies have identified specific causes [51]. Such studies are difficult to realize [47]. And with toxins, controlled trials are unethical.

In 2020, a systematic review of human maternal mycotoxin exposure studies showed none addressed macrocyclic trichothecenes exposure [2]. In 2023, urinary DON levels in second-trimester Chinese pregnant women were associated with lower infant birth weight and size for gestational age [6].

While our high percentages of miscarriages and CAs may be due to the small cohort, they are consistent with trichothecenes’ known toxicity and pathogenic mechanisms, inhibiting protein and nucleic acid synthesis. In animal studies, both DON and T2 cross the placenta [52] and reach embryonal tissues.

Produced by *Stachybotrys* and *Trichoderma*, T2 is a type A trichothecene, as opposed to the type D macrocyclics (e.g., Satratoxin, Riordan E, and Verrucarin A) [53]. T2 toxin is a strong protein and DNA synthesis inhibitor, impairing lymphocyte proliferation, antibody generation, and dendritic cell development [54]. In pregnant mice, oral T2 extracted by ELISA from *Fusarium* and *Trichoderma* cultures caused placental hemorrhage, maternal death, embryo toxicity, fetal loss, and skeletal anomalies [52]. In mice, T2 causes placental bleeding through direct cytotoxic action on fragile vascular structures [54]. It readily passes the placenta into the unborn, causing skeletal and muscle malformations, widespread subcutaneous hematomas, encephalitis, embryonic resorption, and fetal death. Placental hemorrhages have also been attributed to T2’s anticoagulant actions [54].

### 4.3. Clinical Implications

Our data is concerning, and applying the precautionary principle, pregnant and lactating women, as well as neonates, should avoid all contact with indoor environments contaminated with MTs, *Stachybotrys*, or *Trichoderma*. Ideally, they need to be relocated before construction, demolition, or remodeling activities are begun. Exposure to mold-infested buildings and mold-related health complications affecting the unborn are expected to increase with global urbanization, overcrowding, poor living conditions, and global warming [51]. Poverty further enhances indoor mold contamination. In water-damaged buildings, with mold apparent, personal protection is critical. Macrocyclic trichothecenes toxicity is probably cumulative and, in some instances, results in permanent health effects. Unlike the simple trichothecenes, there is no known antidote for MT exposure. Protective gear is needed--including respiratory, eye, and skin protection, as well as disposable gowning covering hair, shoes, and clothing [15]. This is impractical for families, so relocation is usually the best solution. Should MT contamination be found, all items that cannot be decontaminated need to be quarantined or safely discarded.

### 4.4. Research Implications

This study proposes the question of whether moldy indoor conditions are hazardous to the pregnant or lactating mother, the unborn, and the neonate. The hazardous indoor environment is an emerging area for future research involving toxicology, microbiology, medicine, and epidemiology. With the rise in indoor biocontaminant-related disease worldwide, more research is needed into the complexity of additive and synergistic effects from multiple indoor contaminants [55].

Our findings of miscarriages and congenital anomalies in this cohort of women with similar indoor exposures emphasize the urgent need to further develop surveillance methods for collecting real-world clinical data to determine potential risk factors and causal relationships. Parameters for surveillance need to be further defined for this emerging, underrecognized public health threat.

Future research is needed to study indoor mold and mycotoxin exposure health hazards. Studies from contaminated indoor built environments from various geographic locations and climates are needed to achieve true generalizability as well. Human biomonitoring studies are needed wherever significant contamination with *Stachybotrys* or *Trichoderma* is found.

Indoor macrocyclic trichothecenes, *Stachybotrys*, and *Trichoderma* may prove to be reliable indicators for exceptionally hazardous indoor conditions. Airborne *Stachybotrys* spores, unusual in the indoor built environment, may also prove to be a useful marker for an unusually hazardous space and the need for further fungal and MT testing. Practical, affordable, and reliable indoor screening methods need to be developed. Reliable mycotoxin assays and biomarkers are needed for biomonitoring. Placentae and breast milk analysis may prove useful.

### 4.5. Strengths and Limitations

The main strength of this study is in its unique real-world clinical/environmental dataset with timelines of detailed medical observational assessments linked with professional indoor inspection. The clinical evaluation of mothers and babies was thorough, with detailed medical records collected from other practitioners and hospitals where available.

There are several key limitations in this study. One uncertainty relates to the MT ELISA methodology, which only screens for MTs as a group and does not identify or quantify individual trichothecene molecules. Therefore, a positive result cannot reliably distinguish the presence of specific trichothecenes (e.g., satratoxins, roridins, verrucarins). Thus, the presence of other toxins was not excluded, such as T-2 toxin, which could have been involved since both *Stachybotrys* and *Trichoderma* produce it. Confirmation requires more specific analytical methods, such as liquid chromatography-mass spectrometry. Better and validated assays are required, but there were no alternatives available in the USA at the time the testing was done.

Another weakness lies in the standardization of data collection. In each home, data collection was performed by different inspectors, and their observations and measurements were not precisely coincident with exposure. Also, the frequency of mucosal damage in infants is probably underestimated since oronasal examination in small children was difficult, limiting any observations of ulceration or other reasons for symptomatology and poor feeding.

Another weakness is the lag between exposure and medical evaluation, with three (38%) mothers being seen 6–15 years after the initial exposure. Nevertheless, their chronologies and symptomatologies are compellingly similar. All miscarriages occurred within one to two years of moving into odorous, contaminated residences. Moreover, all mothers, except for the nulliparous mother from Home #8, had previous successful pregnancies and births before moving into the contaminated homes. None had a history of any miscarriages either.

## 5. Conclusions

In summary, consistent with in vitro and animal model research, combined with military medicine experience, these epidemiological observations of real-world clinical chronological exposure data and environmental data suggest a direct causal relationship between indoor MT, *Stachybotrys*, or *Trichoderma* exposure and adverse pregnancy and neonatal outcomes. Parameters for surveillance need to be further defined for this emerging public health threat. We hope this report stimulates additional epidemiological work related to water-damaged buildings and pregnancy, as well as the development of improved MT assays and other biomarkers. Further investigation with human biomonitoring is recommended for conditions where *Stachybotrys* or *Trichoderma* are found with environmental and clinical testing, including MTs, done as synchronously as possible. Indoor macrocyclic trichothecenes, *Stachybotrys* and *Trichoderma* may prove to be reliable indicators for exceptionally hazardous indoor conditions. Further research into exposure and reproductive risks, particularly during gestation and lactation, is warranted.

## Data Availability

Data is unavailable due to health information privacy and security regulations.
